# Prediction of nodal disease in oral squamous cell carcinoma of the tongue: histopathological risk assessment with the focus on depth of invasion

**DOI:** 10.1007/s00784-024-05863-4

**Published:** 2024-08-06

**Authors:** Friedrich Mrosk, Viktor Krom, Christian Doll, Lukas Mödl, Kilian Kreutzer, Jan Voss, Carsten Rendenbach, Max Heiland, Steffen Koerdt

**Affiliations:** 1 Charité – Universitätsmedizin Berlin, Corporate Member of Freie Universität Berlin and Humboldt- Universität zu Berlin, Department of Oral and Maxillofacial Surgery, Augustenburger Platz 1, 13353 Berlin, Germany; 2 Charité – Universitätsmedizin Berlin, Corporate Member of Freie Universität Berlin and Humboldt- Universität zu Berlin, Institute of Biometry and Clinical Epidemiology, Charitéplatz 1, 10117 Berlin, Germany; 3 Berlin Institute of Health at Charité – Universitätsmedizin Berlin, Charitéplatz 1, 10117 Berlin, Germany

**Keywords:** Oral squamous cell carcinoma, Cervical lymph node metastasis, Depth of invasion, Prediction

## Abstract

**Objective:**

Cervical lymph node metastasis (CLNM) is one of the most relevant influencing factors for the oncological outcome of patients with oral squamous cell carcinoma (OSCC). Several studies showed that the tumors depth of invasion (DOI) influences the risk for CLNM, however varying across the oral subsites. The aim of this study is to investigate the role of DOI and other risk factors in OSCC of the tongue in relation to the occurrence of occult CLNM.

**Materials and methods:**

In this retrospective study, *n* = 139 patients with primary OSCC of the tongue, treated by complete surgical resection (R0) with curative intention between 2013 and 2021, were included. For data analysis, epidemiologic data as well as preoperative tumor staging, surgical therapy including neck management, histopathological tumor data and follow-up were considered. Uni- and multivariate logistic regression were used to determine association between histopathological risk factors and the occurrence of occult CLNM.

**Results:**

The rate of occult cervical metastasis was 19.4%. T-staging, cervical nodal disease (pN+) and lymphatic invasion were significantly associated with reduced OS and RFS. While DOI had no relevant influence on the OS and RFS (*p* = 0.88 and *p* = 0.91 respectively), there was significant correlation between DOI and the occurrence of occult CLNM (OR: 1.17, 95%CI: 1.05–1.30; *p* < 0.01). The optimal cutoff in predicting occult CLNM was 6 mm (Sensitivity: 84.2%, Specificity: 73.5%, AUC: 0.75).

**Conclusions:**

The DOI is a helpful risk parameter to predict the occurrence of occult nodal disease in OSCC of the tongue. Given the critical decision cutoff between 2 and 4 mm DOI for performing elective neck dissection in the current guidelines, our data suggests that in these cases, surgical de-escalation could be feasible with close follow-up.

**Clinical relevance:**

This study highlights the relevance of DOI as a risk parameter in the prediction of CLNM with the aim to specify the individual patient risk and to deescalate surgical therapy in order to decrease comorbidities while improving the oncological prognosis.

## Introduction

Cervical lymph node metastasis (CLNM) is one of the most relevant influencing factors determining the oncological outcome of patients with oral squamous cell carcinoma (OSCC). Even in the clinically unsuspicious neck (cN0), the probability of occult CLNM remains high at 23 – 45% [1, 2]. Therefore, neck management is generally recommended in current guidelines [3, 4]. Although elective neck dissection (END) is still considered the standard of care in most countries for the cN0 neck, deescalating procedures such as sentinel lymph node biopsy (SLNB) are becoming increasingly popular especially for early-stage disease to avoid overtreatment [5–7]. In addition, new advances in drug-based tumor therapy, especially immunotherapy in the neoadjuvant setting, may play a role in the near future [8].

There is therefore great interest in predicting CLNM as reliably as possible. Several histopathologic risk factors have been studied that can predict CLNM, such as tumor size, histological grading, and depth of invasion (DOI) [9]. In 2017, DOI was included as an additional criteria for T-staging in the current TNM-classification of the American Joint Commission on Cancer (AJCC) [10]. Several studies have investigated the role of DOI in relation to oncological outcomes, as well as as a risk predictor for CLNM [11–15]. Nevertheless, the suggested cutoff values vary among study cohorts, especially between tumor subsites. As a result, recommended DOI cutoffs to guide optimal neck management, especially whether to perform END, have been kept rather low. In the current NCCN guidelines, the choice of whether to perform an END can be weighted based on DOI between 2 and 4 mm and above [4]. It is a matter of debate whether individual tumor subsites have differences in terms of the prognosis and risk profile [16]. The tongue is one of the most frequently affected subsites for OSCC and the oncological prognosis seems to remain poor there [17]. Furthermore, studies have shown that OSCC of the tongue is associated with higher prevalence of occult CLNM, higher probability of level IV/V metastasis, as well as contralateral nodal disease [18–20]. Especially small tumors are of particular clinical interest: since some cases may allow total excision in the context of excision biopsies, guided neck treatment becomes clinically relevant. There is therefore particular interest in further characterizing the risk profile of this subsite to guide clinical decision making. The aim of this study was to investigate the role of DOI in the tongue subsite in relation to the risk of occult CLNM.

## Methods

### Study design

This retrospective study was conducted in accordance with the applicable ethical guidelines and was approved by the institutional ethics committee (EA2/077/20). All patients with primary OSCC of the tongue, treated by complete surgical resection with curative intent between 2013 and 2021 in the Department of Oral and Maxillofacial Surgery at the Charité – Universitätsmedizin Berlin, Germany, were assessed and screened for inclusion. Patients with incomplete resection and concomitant carcinoma, presence of distant metastases at the time of primary treatment or SCC of the base of the tongue were excluded. All patient data regarding epidemiology, preoperative tumor staging, surgical therapy including neck management, histopathological tumor data, and follow-up were retrospectively reviewed from the patients’ charts. All cases treated prior to 2017 were restaged according to the recent AJCC Cancer Staging Manual, 8th Edition [10]. Neck dissection was performed either as selective neck dissection (SND) extending to the cervical levels I-III or comprehensive neck dissection (CND) also including the caudally located levels IV-V with the preservation of the sternocleidomastoid muscle, the internal jugular vein and the spinal accessory nerve according to Robbins et al. [4, 21]. Furthermore, patients received adjuvant treatment if indicated and according to the current guideline recommendations [4].

### Depth of invasion

At our institute, DOI is measured in a standardized fashion according to the recent AJCC Cancer Staging Manual, vertically to the deepest point of infiltration from the level of the basal membrane of the closest adjacent regular tissue of the oral mucosa [10]. DOI was recorded in millimeters.

### Statistical analysis

Metric data was presented including mean values ± standard deviation as well as median with interquartile ranges (IRQ: 25th − 75th percentile). Univariate and multivariate logistic regression analysis was performed to determine associations between histopathological risk factors and the occurrence of occult nodal disease. Receiver-operating curves (ROC) were generated to determine cutoff values by the Youden index for significant correlations. The area under the curve (AUC) is used to describe the model’s performance. The parameter DOI was introduced as increments of 1 mm to the statistical analyses. Overall survival (OS) and the recurrence free survival (RFS) were determined through Kaplan-Meier analysis, including 95% CIs. Survival curves were statistically analyzed using the log rank test. Only the statistically significant variables from univariate analyses were selected for the multivariate analyses. P-values of < 0.05 were considered to be statistically significant. All statistical analyses were conducted using SPSS version 28 (IBM Corp., USA).

## Results

### Patient characteristics

Overall, 139 patients could be included. Age ranged between 24 and 92 years (median 63, IQR: 54–74). Risk factors, such as tabacco and/or alcohol consumption, were present in 92 (50.3%) cases. Preoperative clinical T-staging was as follows: cT1 in 66 (47.5%) cases, cT2 in 61 (48.6%) cases, cT3 in 10 (7.2%) cases and cT4a in 2 (1.4%) cases. Patient’s characteristics and histopathological results are presented in Table 1.

### Neck management and adjuvant treatment

Selective neck dissection (SND) was performed in 108 (77.7%) cases while 13 (9.4%) patients received comprehensive neck dissection (CND) and 18 (12.9%) patients SLNB without completion neck dissection due to negative sentinel nodes. In addition, 128 (92.1%) patients were treated unilaterally while 11 (7.9%) patients received bilateral ND. Mean lymph node yield (LNY) was 24.0 (± 10.7), ranging between 11 and 78. In cases of pN+, the mean number of affected lymph nodes was 2.0 (± 1.2), ranging between 1 and 5. Furthermore, the mean lymph node ratio (LNR) was 8.4% (± 5.8), ranging between 2.6 and 30.0%.

Adjuvant therapy (AT) was carried out in 26 cases (18.7%). From these, 14 (53.8%) received postoperative radiotherapy (PORT) alone while 12 (46.2%) received postoperative radiochemotherapy (PORCT).

### Histopathological assessment

The distribution of early staged diseases (pT1-2) versus advances stage diseases (pT3) was 88.5% versus 11.5%, respectively. Furthermore, the rate of occult metastasis was 19.4% in this study cohort. DOI ranged from 0.5 mm to 35 mm (mean 7.3 mm ± 5.8 mm). The correlation coefficient between pT-stage and DOI was 0.77. From 27 patients presenting with positive nodal disease, 46 metastases were detected. The distribution to the cervical levels were as follows: Ia: 0 (0%); Ib: 3 (6.5%); IIa: 10 (21.7%); IIb: 4 (8.7%), III: 27 (58.7%); IV: 1 (2.2%); V: 1 (2.2%).

**Table 1:** Histopathological results

	Overall (*n* = 139)
**Age** (median, IQR)	63 (54–74)
**Sex**	
male	78 (56.1
female	61 (43.9)
**pT-stage (%)**	
pT1	68 (48.9)
pT2	55 (39.6)
pT3	16 (11.5)
pT4	-
**pN-stage (%)**	
pN0	112 (80.6)
pN1	11 (7.9)
pN2a	3 (2.2)
pN2b	2 (1.4)
pN2c	-
pN3	11 (7.9)
**Disease stage**	
Stage I	65 (46.8)
Stage II	43 (30.9)
Stage III	15 (10.8)
Stage IVa	10 (7.2)
Stage IVb	6 (4.3)
**Extracapsular spread**	
yes	11 (7.9)
no	128 (92.1)
**Close margins**	
≤ 5 mm	22 (15.8)
> 5 mm	117 (84.2)
**Vascular invasion**	
yes	2 (1.4)
no	137 (97.1)
**Lymphatic invasion**	
yes	4 (2.9)
no	135 (97.1)
**Perineural invasion**	
yes	9 (6.5)
no	130 (93.5)
**Grade of differentiation**	
G1	18 (12.9)
G2	109 (78.4)
G3	12 (8.6)
**Depth of invasion** (mean ± SD)	6.7 ± 5.7

### Risk prediction of occult cervical nodal disease

**Fig. 1:**
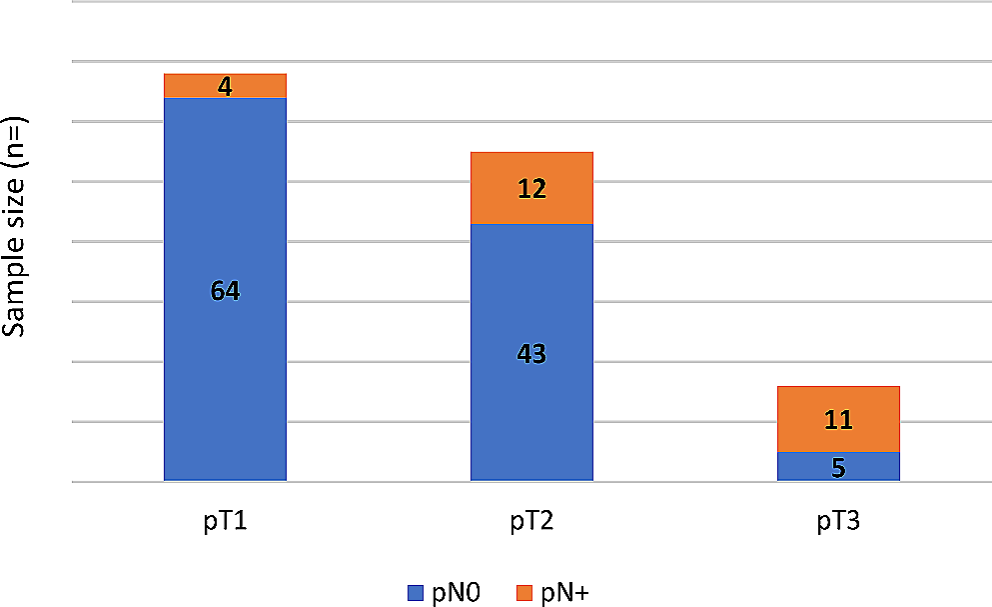
Prevalence of occult nodal disease among the pT-staging (*p* < 0.01)

The univariate logistic regression model for the prediction of occult nodal disease is shown in Table 2. The distribution of occult metastasis among the pT-stage is shown in Fig. 1.

**Table 2:** Univariate logistic regression analysis for occult nodal disease

	OR	95% CI	*p*-value
Age	1.02	0.99–1.05	0.28
Sex	1.17	0.50–2.75	0.71
pT-stage	3.84	2.13–6.92	< 0.01
Depth of invasion	1.17	1.05–1.30	< 0.01
Vascular invasion	1.46	0.13–16.44	0.76
Lymphatic invasion	1.40	0.14–13.98	0.78
Perineural invasion	6.14	1.53–24.70	0.01
Grade of differentiation	5.69	1.95–16.63	< 0.01

In multivariate analysis, pT-stage (OR: 2.99; 95%CI: 1.56–5.71; *p* < 0.01) and perineural invasion (OR: 5.39; 95%CI: 1.07–27.17; *p* = 0.04) significantly correlated with the occurrence of nodal disease.

### Oncological outcome and disease recurrence

Follow-up ranged from 8 to 136 months (median 42 months, IQR: 25–65). During the follow-up period, 23 (16.5%) patients presented with disease recurrence. From these, local disease recurrence was present in 9 (6.5%) patients while secondary CLNM (regional recurrence) was present in 12 (8.6%) and distant metastasis was present in 8 (5.8%) patients. Furthermore, 17 (12.2%) patients died during follow-up.

The overall 5-year OS was 85.2% [95%CI: 77.8–92.6]. The following histopathological risk factors had a statistically significant influence on the 5-year OS: pT-Stage (pT1-3: 91.5% [95%CI: 83.5–99.5], 82.5% [95%CI: 73.1–91.9] and 67.0% [95%CI: 54.1–79.9], respectively; *p* < 0.01), pN+ (65.4% [95%CI: 46.4–84.4] versus 89.4% [95%CI: 82.4–96.4]; *p* < 0.01) and lymphatic invasion (37.5% [95%CI: 23.8–51.2] versus 87.1% [95%CI: 80.1–94.1]; *p* = 0.04).

**Fig. 2:**
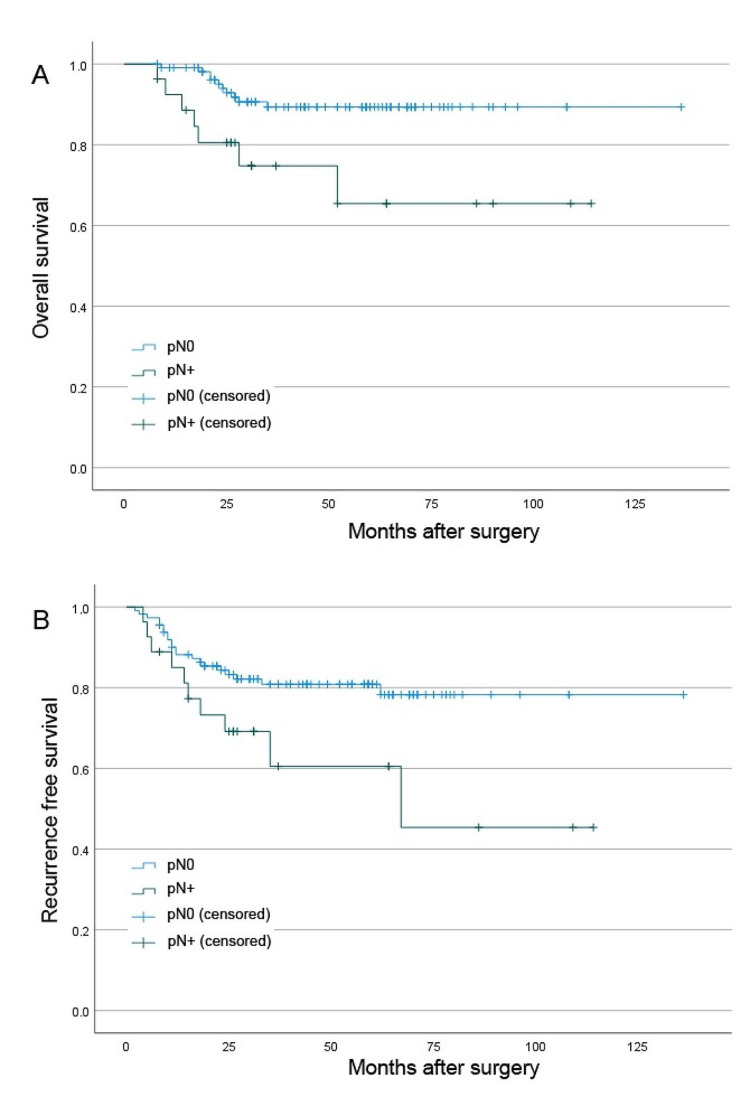
Kaplan Meier survival analysis showing the (**A**) overall survival and (**B**) the recurrence free survival of the overall cohort in relation to cervical nodal disease

The overall 5-year RFS was 75.3% [95%CI: 65.6–85.0]. There were no statistically significant differences between the pT-stages regarding 5-year RFS (*p* = 0.14). However, patients with pT3 stage presented with lower RFS as compared to pT1-2 (61.1% [95%CI: 47.2–75.0] versus 77.2% [95%CI: 67.1–87.3], *p* = 0.08). The 5-year RFS was significantly lower in cases of pN+ (60.5% [95%CI: 38.8–82.2] versus 78.3% [95%CI: 68.5–88.1], *p* = 0.03), as seen in Fig. 2.

### The influence of the depth of invasion

**Fig. 3:**
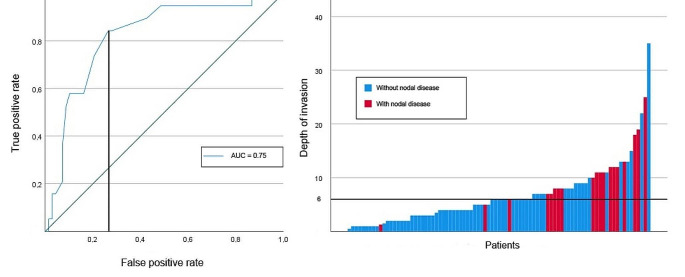
Predictive performance of depth of invasion for occult nodal disease (**A**: area under the receiving operator curve for the logistic regression with the Youden index. **B**: distribution of each patient and the depth of invasion regarding the presence of occult nodal disease in relation to the selected cut-off.)

In this study, DOI was significantly higher in cases of pN+ (mean 10.8 mm ± 5.4 versus mean 5.6 mm ± 5.2; *p* < 0.01). In addition, DOI was significantly associated with the occurrence of occult nodal disease (OR: 1.17, 95%CI: 1.05–1.30; *p* < 0.01). The model was able to achieve a fair separation of DOI based on pN+ (AUC: 0.75), as presented in Fig. 3. The optimal cutoff for predicting occult CLNM was 6 mm, with a sensitivity of 84.2% and specificity of 73.5%. Below the 6 mm cutoff, 3 (2.2%) patients presented with occult nodal disease.

There was no significant relationship between DOI and the occurrence of disease recurrence during follow-up (OR: 1.06, 95%CI: 0.99–1.14; *p* = 0.12). In the survival analysis, the DOI cutoff had neither statistically significant nor clinically relevant influence on 5-year OS (90.1% versus 83.9%, *p* = 0.88) and 5-year RFS (81.3% versus 78.1%, *p* = 0.91).

## Discussion

In recent years, early staged, node-negative OSCCs (pT1-2pN0) have been a focus of discussion, as de-escalation from END to SLNB can be considered in these cases.

The current NCCN guidelines state that END can be considered in the case of a DOI between 2 and 4 mm, while values below 2 mm should be highly selective situations for END compared to SLNB or watch and wait [4]. The reference for the current guidelines regarding this cutoff is a prospective study from D`Cruz et al., which reported a potential lack of benefit of END with 3 mm or less of DOI [22]. It is therefore of great clinical interest to predict these situations where END can be avoided to deescalate lymph node treatment. Nevertheless, END appears to be superior in terms of oncological prognosis compared to watch and wait of patients with tongue carcinoma [23]. Over the recent years, SLNB became increasingly more relevant for the treatment of OSCC [24]. For head and neck melanoma, the Second Multicenter Selective Lymphadenectomy Trial (MSLT-II) even caused for a paradigm shift regarding completion neck dissection after positive SLN [25]. The trial showed that completion neck dissection offered improved regional control while the disease-specific survival was not increased as compared to observation only. Furthermore, new advances in immunotherapy become more and more relevant, not only in an adjuvant setting but also with potential benefits as neoadjuvant treatment modalities influencing the need for surgical lymph node intervention [26]. However, these novel approaches still need validation for head and neck squamous cell carcinoma and OSCC in particular. Several studies have investigated the influence of DOI in relation to the occurrence of nodal disease and oncological outcome with a fixed cutoff value [12, 27–29]. Tan et al. and Wu et al. reported significant differences in OS and RFS below a DOI cutoff of 4 mm [27, 28]. In the study by Muhammad et al. with a set cutoff of 5 mm, the authors also reported significantly more occult nodal disease above the DOI cutoff [29]. However, these studies did not determine a specific cutoff value for their cohort. In 1994, Weiss et al. published a study in which the authors analyzed the management strategy for the cN0-neck in terms of END versus observation only using a decision tree model [30]. The authors concluded that above a probability of 20% of occult nodal disease, treatment of the neck would be warranted. This 20% threshold is among others that are also part of the current ASCO practical guidelines [31]. This threshold was also a cutoff strategy for determining the ideal DOI for OSCC in the literature [32, 33]. Here, Aaboubout et al. reported the optimal DOI cutoff of 4.3 mm across all tumor subsites [32]. In a study by Feng et al., the tumor subsites were analyzed separately, giving cutoff values of 2 mm for the tongue, 3 mm for the floor of the mouth and upper gingiva, and 4 mm for the lower gingiva [33]. These subsite differences could also be shown for the OSCC of the upper oral cavity [15]. Using the minimum p value method for tongue OSCC, Liu et al. determined a 4.5 mm DOI cutoff [34]. Also using the ROC method, den Toom et al. reported a relatively low DOI cutoff value of 3.4 mm across all tumor subsites for predicting occult nodal disease [13]. In contrast to these findings, Tam et al. determined a 7.25 mm DOI cutoff also using the ROC method, which is similar to the findings of our study [14].

As well as in the prediction of occult nodal disease, several studies have also shown that DOI had an influence on oncological outcome in terms of OS and RFS. In a study of Newman et al., pT3 and a DOI above 10 mm were associated with significantly worse survival [35]. In our study, a significant association between DOI and OS as well as RFS could not be shown. However, patients with pT3 tumors also had worse survival, which can be indirectly transferred to DOI above 10 mm. Another study reported that DOI predicted occult nodal disease, but only the pT stage had a significant influence on the survival [36]. In one recent meta study, predicting nodal disease was the most common finding throughout all assessed studies independent of any cutoff; however, there was low certainty of evidence [37]. Furthermore, the effect on the survival was more inconsistent.

Determining the DOI of tumors and knowing the optimal cutoff for predicting occult nodal disease before primary treatment may help to guide clinical decision making. One point of criticism in the current discussion is that the DOI is usually obtained after primary resection where neck management has already occurred. Several studies have shown promising results from ultrasound imaging techniques, while MRI imaging consistently overestimated the DOI [38–40]. However, preoperatively assessing the DOI to guide the clinical decision-making process still requires validation from prospective studies and is so far not a part of current guidelines.

For this study, several limitations must be noted. Firstly, significant associations between risk parameters and the prediction of occult metastasis or survival may be object to a survivorship bias. Moreover, the estimated associations may be biased due to the omission of relevant variables, which are not directly observable in the data but may influence the relationship between the observed variables. In addition, the results of this study are limited by a small sample size of histopathological risk factors. Therefore, statistically significant associations between these events must be interpreted with caution. It should be noted that the DOI is usually correlated with the pT stage due to its classification systematic. Therefore, some degree of multicollinearity is expected to be present in our models. Multicollinearity shouldn’t bias the parameter estimates themselves, but it inflates the standard errors of the affected variables, making their estimated coefficients less precise and potentially numerically unstable. However, we believe that a correlation of 0.77 between pT and DOI is still acceptable from a numerical point of view. We determined that a DOI threshold of 6 mm exhibited optimal performance according to the Youden criteria. Nonetheless, it is crucial to recognize that this threshold’s optimality is subject to the specific data set employed. Consequently, one should be cautious when generalizing this finding. From the 27 patients with CLNM, 24 had a DOI above 6 mm, which is a difference of 8.9%. Therefore, fully relying on this threshold may result in false negative cases.

## Conclusion

In this study, we were able to show that in OSCC of the tongue, DOI is significantly associated with the occurrence of occult nodal disease. The optimal threshold of DOI according to the Youden index was 6 mm. There was no significant association between DOI and the OS and RFS. The critical decision cutoff is between 2 and 4 mm DOI for performing END in the NCCN guidelines: our data suggests that in these cases, de-escalation may be feasible with close follow-up.

## Data Availability

No datasets were generated or analysed during the current study.
