# Correction to: Prediction of nodal disease in oral squamous cell carcinoma of the tongue: histopathological risk assessment with the focus on depth of invasion

**DOI:** 10.1007/s00784-025-06469-0

**Published:** 2025-07-28

**Authors:** Friedrich Mrosk, Viktor Krom, Christian Doll, Lukas Mödl, Kilian Kreutzer, Jan Voss, Carsten Rendenbach, Max Heiland, Steffen Koerdt

**Affiliations:** 1 Department of Oral and Maxillofacial Surgery, Charité– Universitätsmedizin Berlin, Corporate Member of Freie Universität Berlin and Humboldt- Universität zu Berlin, Augustenburger Platz 1, 13353 Berlin, Germany; 2 Charité– Universitätsmedizin Berlin, Corporate Member of Freie Universität Berlin and Humboldt- Universität zu Berlin, Institute of Biometry and Clinical Epidemiology, Charitéplatz 1, 10117 Berlin, Germany; 3 Berlin Institute of Health at Charité– Universitätsmedizin Berlin, Charitéplatz 1, 10117 Berlin, Germany

**Correction to: Clinical Oral Investigations**

10.1007/s00784-024-05863-4

The article “Prediction of nodal disease in oral squamous cell carcinoma of the tongue: histopathological risk assessment with the focus on depth of invasion”, written by Friedrich Mrosk, Viktor Krom, Christian Doll, Lukas Mödl, Kilian Kreutzer, Jan Voss, Carsten Rendenbach, Max Heiland and Steffen Koerdt, was originally published Online First without Open Access. After publication in volume 28, issue 9, pages 1–8 the author decided to opt for Open Choice and to make the article an Open Access publication. Therefore, the copyright of the article has been changed to © The Author(s) 2025 and the article is forthwith distributed under the terms of the Creative Commons Attribution 4.0 International License, which permits use, sharing, adaptation, distribution and reproduction in any medium or format, as long as you give appropriate credit to the original author(s) and the source, provide a link to the Creative Commons licence, and indicate if changes were made. The images or other third party material in this article are included in the article’s Creative Commons licence, unless indicated otherwise in a credit line to the material. If material is not included in the article’s Creative Commons licence and your intended use is not permitted by statutory regulation or exceeds the permitted use, you will need to obtain permission directly from the copyright holder. To view a copy of this licence, visit http://creativecommons.org/licenses/by/4.0.

The original article has been corrected.

